# Investigating multidrug efflux pumps associated with fatty acid salt resistance in *Escherichia coli*

**DOI:** 10.3389/fmicb.2023.954304

**Published:** 2023-02-21

**Authors:** Seiji Yamasaki, Tomohiro Yoneda, Sota Ikawa, Mitsuko Hayashi-Nishino, Kunihiko Nishino

**Affiliations:** ^1^SANKEN (The Institute of Scientific and Industrial Research), Osaka University, Osaka, Japan; ^2^Graduate School of Pharmaceutical Sciences, Osaka University, Osaka, Japan; ^3^Institute for Advanced Co-Creation Studies, Osaka University, Osaka, Japan; ^4^Center for Infectious Disease Education and Research, Osaka University, Osaka, Japan

**Keywords:** bacteria, *Escherichia coli*, fatty acid salts, multidrug efflux pump, resistance

## Abstract

Fatty acids salts exert bactericidal and bacteriostatic effects that inhibit bacterial growth and survival. However, bacteria can overcome these effects and adapt to their environment. Bacterial efflux systems are associated with resistance to different toxic compounds. Here, several bacterial efflux systems were examined to determine their influence on fatty acid salt resistance in *Escherichia coli.* Both *acrAB* and *tolC E. coli* deletion strains were susceptible to fatty acid salts, while plasmids carrying *acrAB*, *acrEF*, *mdtABC*, or *emrAB* conferred drug resistance to the Δ*acrAB* mutant, which indicated complementary roles for these multidrug efflux pumps. Our data exemplify the importance of bacterial efflux systems in *E. coli* resistance to fatty acid salts.

## Introduction

Fatty acid salts exhibit amphipathic properties and antibacterial activities which are mediated by (i) increased membrane permeability and leakage, (ii) disrupted electron transport chain and oxidative phosphorylation uncoupling, and (iii) inhibited nutrient uptake and membrane enzyme activity (Yoon et al., 2018). While bacteria employ several strategies to resist the antibacterial actions of fatty acid salts (Miller et al., 1977; Chamberlain et al., 1991; Desbois and Smith, 2010), the resistance mechanisms remain unclear, therefore, it is important to comprehend how bacteria evade/abrogate the bactericidal effects of fatty acid salts.

Bacterial drug resistance is associated with drug efflux pumps which reduce drug accumulation in cells (Nikaido, 1996; Zgurskaya and Nikaido, 2000). These pumps are classified into six categories based on sequence similarity with other protein families: major facilitator superfamily (MFS), resistance-nodulation-cell division (RND), small multidrug resistance (SMR), multidrug and toxic compound extrusion (MATE), ATP-binding cassette (ABC), and proteobacterial antimicrobial compound efflux families (PACE; Putman et al., 2000; Hassan et al., 2018). The elucidation of bacterial genome sequences has greatly facilitated the identification of putative drug resistance genes in Gram-negative bacteria, including *Escherichia coli* (Nishino and Yamaguchi, 2001). Of note, the RND family has a major role in both intrinsic and acquired multidrug resistance in Gram-negative bacteria (Venter et al., 2015). RND efflux pumps require two proteins to function: a membrane fusion protein (periplasmic adaptor protein) and an outer membrane protein, e.g., the major drug efflux pump AcrB of the RND family requires the membrane fusion protein AcrA and the outer membrane protein TolC to function (Nishino et al., 2003; Alav et al., 2021; Zwama and Nishino, 2021).

In some bacteria, multidrug efflux pumps are believed to play vital functions overcoming the antibacterial effects of fatty acid salts (Ma et al., 1995; Gunn, 2000; Rosenberg et al., 2003; Prouty et al., 2004; Lennen et al., 2013; Wotzka et al., 2019; Henderson et al., 2021; Yoneda et al., 2022). In this study, we evaluated multidrug efflux pump functions toward fatty acid salt resistance using various *E. coli* strains deficient in or overexpressing genes encoding multidrug efflux pumps. Using this strategy, we identified multidrug efflux pumps and mechanisms involved in bacterial resistance to fatty acid salts.

## Materials and methods

### Strains and plasmids used in this study

Study strains and plasmids are shown (Table 1). *Escherichia coli* strains were derived from the MG1655 wild-type (WT) strain (Blattner et al., 1997). To construct *E. coli* gene deletion mutants, gene disruption strategies were performed according to Datsenko and Wanner (2000) using the primers listed in Table 2. Plasmids carrying *acrAB*, *acrD*, *acrEF*, *mdtABC*, *mdtEF*, *emrAB*, *macAB*, *emrE*, *mdfA*, or *mdtK* were constructed as previously described (Nishino et al., 2003).

**Table 1:** *Escherichia coli* strains used in this study.

*Escherichia coli* strains	Characteristics	Source or reference
MG1655	*Escherichia coli* wild-type	Blattner et al. (1997)
NKE3002	Δ*emrAB*::Km^R^	This study
NKE3003	Δ*emrAB*::Km^R^/vector (pHSG398)	This study
NKE3004	Δ*emrAB*::Km^R^ /p*emrAB*	This study
NKE3005	Δ*emrAB*::Km^R^Δ*tolC*	This study
NKE3006	Δ*emrAB*::Km^R^Δ*tolC*/vector (pHSG398)	This study
NKE3007	Δ*emrAB*::Km^R^Δ*tolC*/p*emrAB*	This study
NKE348	Δ*acrAB*	Nishino et al. (2003)
NKE95	Δ*tolC*::Cm^R^	This study
NKE128	Δ*acrAB*Δ*tolC*	This study
NKE348	Δ*acrAB*	Nishino et al. (2003)
NKE473	Δ*acrAB*/vector (pHSG399)	Nishino et al. (2003)
NKE386	Δ*acrAB*/p*acrAB*	Nishino et al. (2003)
NKE388	Δ*acrAB*/p*acrD*	Nishino et al. (2003)
NKE390	Δ*acrAB*/p*acrEF*	Nishino et al. (2003)
NKE391	Δ*acrAB*/p*mdtABC*	Nishino et al. (2003)
NKE474	Δ*acrAB*/p*mdtEF*	Nishino et al. (2003)
NKE393	Δ*acrAB*/p*emrAB*	Nishino et al. (2003)
NKE395	Δ*acrAB*/p*macAB*	Nishino et al. (2003)
NKE397	Δ*acrAB*/p*emrE*	Nishino et al. (2003)
NKE396	Δ*acrAB*/p*mdfA*	Nishino et al. (2003)
NKE399	Δ*acrAB*/p*mdtK*	Nishino et al. (2003)

**Table 2:** Primers used in this study.

Primer	Sequence (5′–3′)
*emrA*-P1	TCGGCTCAGCCGATGAGTTAAGAAGATCGTGGAGAACAATGTGTAGGCTGGAGCTGCTTC
*emrB*-P2	ATTGAAAAAAGCCAGTTCAAATGAACTGGCTTAGTTGTACCATATGAATATCCTCCTTAG
*tolC*-P1	ACTGGTGCCGGGCTATCAGGCGCATAACCATCAGCAATAGGTGTAGGCTGGAGCTGCTTC
*tolC*-P2	TTACAGTTTGATCGCGCTAAATACTGCTTCACCACAAGGACATATGAATATCCTCCTTAG

### Measurement of the minimum inhibitory concentrations of fatty acid salts

To examine multidrug efflux pumps in *E. coli* fatty acid salt resistance, antibacterial activities were examined on LB plates containing sodium hexanoate (C6) at concentrations from 78 to 40,000 μg/ml, sodium octanoate (C8) at concentrations from 78 to 40,000 μg/ml, sodium decanoate (C10) at concentrations from 0.63 to 40,000 μg/ml, and sodium dodecanoate (C12) at concentrations from 0.08 to 5,000 μg/ml (Sigma-Aldrich, St Louis, MO, United States; Table 3). Plates were prepared using the 2-fold agar dilution technique (Nishino and Yamaguchi, 2004). To determine minimum inhibitory concentrations (MICs), bacteria were cultured overnight at 37°C in LB, diluted in the same medium, and tested at a final inoculum of 10^5^ colony forming units/spot using McFarland turbidity standards (Eiken Chemical, Tokyo, Japan) and a multipoint inoculator (Sakuma Seisakusyo, Tokyo, Japan). Plates were incubated at 37°C for 20 h.

**Table 3:** *Escherichia coli* strain susceptibility to sodium hexanoate (C6), sodium octanoate (C8), sodium decanoate (C10), and sodium dodecanoate (C12) using minimum inhibitory concentrations (MIC).

	MIC (μg/ml)			
	C6	C8	C10	C12
Wild-type	20,000	10,000	10,000	>5,000
Δ*emrAB*	10,000	5,000	5,000	5,000
Δ*emrAB*/vector	10,000	5,000	5,000	5,000
Δ*emrAB*/p*emrAB*	10,000	**10,000**	**10,000**	**>5,000**
Δ*emrAB*Δ*tolC*	5,000	625	39	10
Δ*emrAB*Δ*tolC*/vector	5,000	625	39	10
Δ*emrAB*Δ*tolC*/p*emrAB*	5,000	625	39	10
Δ*tolC*	5,000	625	39	10
Δ*acrAB*Δ*tolC*	5,000	625	39	10
Δ*acrAB*	5,000	5,000	1,250	625
Δ*acrAB*/vector	5,000	5,000	1,250	625
Δ*acrAB*/p*acrAB*	5,000	5,000	**2,500**	**2,500**
Δ*acrAB*/p*acrD*	5,000	5,000	1,250	**1,250**
Δ*acrAB*/p*acrEF*	5,000	5,000	1,250	**2,500**
Δ*acrAB*/p*mdtABC*	**10,000**	**10,000**	**2,500**	**2,500**
Δ*acrAB*/p*mdtEF*	5,000	5,000	1,250	**1,250**
Δ*acrAB*/p*emrAB*	5,000	5,000	**2,500**	**5,000**
Δ*acrAB*/p*macAB*	5,000	5,000	1,250	**1,250**
Δ*acrAB*/p*emrE*	**10,000**	5,000	1,250	**1,250**
Δ*acrAB*/p*mdfA*	5,000	5,000	1,250	**1,250**
Δ*acrAB*/p*mdtK*	5,000	5,000	1,250	**1,250**

MICs were determined from at least three repeated measurements. Values in bold greater than those of the parental strains harboring the vector.

### Measurement of the bacterial growth in the presence of sodium dodecanoate (C12)

Single *E. coli* colonies were inoculated into 2 ml of LB. Bacteria were cultured overnight at 37°C, diluted in the same medium, and tested at a final inoculum concentration of 10^5^ colony forming units/μl in 200 μl of LB broth containing sodium dodecanoate (C12; 1,000 μg/ml) using McFarland turbidity standards (Eiken Chemical, Tokyo, Japan). Then, liquid cultures were incubated and shaken at 37°C in NUNC Edge 96-well plates (Thermo Scientific, MA, United States). Bacterial growth was measured by OD_600nm_ readings using the Infinite M200 PRO plate reader (Tecan, Männedorf, Switzerland).

## Results and discussion

In order to investigate the involvement of multidrug efflux pumps in the fatty acid salt susceptibility of *E. coli*, MIC measurements were carried out as described in the section materials and methods. Strains lacking *acrAB* (coding for the bacterial efflux system) or *tolC* (outer membrane channel gene) were more susceptible to fatty acid salts when compared with the WT strain (Table 3). These observations showed that the antibacterial activity of fatty acid salts increased with carbon atom numbers in salts, i.e., MIC values for Δ*tolC* in *E. coli* decreased as carbon atoms increased (Table 3). Both Δ*tolC* and Δ*acrAB*Δ*tolC* mutants were more sensitive to sodium octanoate (C8), sodium decanoate (C10), and sodium dodecanoate (C12) than the Δ*acrAB* mutant, suggesting TolC was needed for fatty acid salt resistance as it functioned not only with AcrAB but also with other multidrug efflux pumps in a protein complex in *E. coli* (Zgurskaya et al., 2011; Lennen et al., 2013).

**Figure 1:**
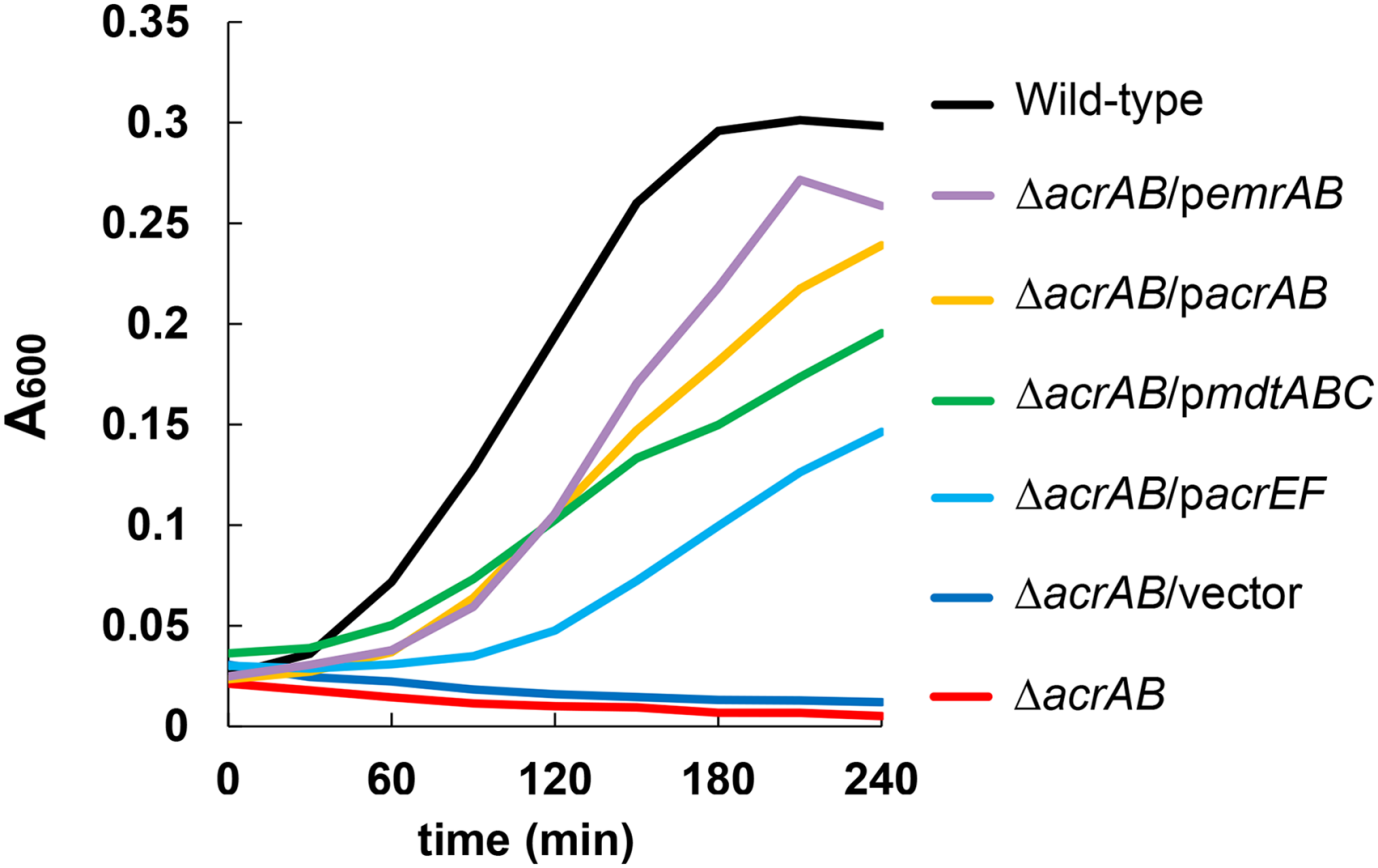
Effects of multidrug efflux pumps on the growth of *Escherichia coli* in the presence of 1,000 μg/ml of sodium dodecanoate (C12). The growth of the *acrAB* mutants with plasmids carrying multidrug efflux pump genes. One of the three experiments that have the similar results is shown.

Furthermore, plasmids carrying drug efflux pump genes belonging to RND (*acrAB*, *acrD*, *acrEF*, *mdtABC*, and *mdtEF*), MFS (*emrAB* and *mdfA*), ABC (*macAB*), SMR (*emrE*), and MATE (*mdtK*) families were transformed into the Δ*acrAB E. coli* strain, and susceptibility to fatty acid salts measured (Table 3). When *acrAB*, *acrEF*, *mdtABC*, or *emrAB* were overexpressed in the Δ*acrAB* strain, a 4–8-fold increase in resistance to sodium dodecanoate (C12) was observed. The complementation of *acrAB* on the plasmid in the Δ*acrAB* strain did not completely reconstitute the resistance of the wild-type strain. This is often the case with some antimicrobial compounds, since the expression of *acrAB* in the wild-type strain is quite high (Nishino et al., 2003). In particular, *emrAB* expression conferred the highest resistance level (Table 3) and the fastest growth rate (Figure 1). Thus, multiple efflux pumps had complementary roles with AcrAB in generating fatty acid salt resistance in *E. coli*.

Our finding that AcrAB belonging to the RND family is major efflux pump to contribute the resistance to fatty acid salts is consistent with previous reports (Rosenberg et al., 2003; Lennen et al., 2013). In a previous report provided by Rosenberg et al., they showed the mechanism of *acrAB* induction by decanoate is mediated with Rob (XylS/AraC family regulator). In future, the detailed mechanism of fatty acid salts resistance we found should be further investigated. Additionally, we could demonstrate that the efflux pump EmrAB, a class of MFS, was specifically involved in fatty acid salt sensitivity in *E. coli*. Of note, considering the previous findings, EmrAB is one of pumps to complement with AcrAB across Gram-negative bacteria, including *E. coli* and *Salmonella enterica* (Lennen et al., 2013; Yoneda et al., 2022). In *S. enterica*, EmrAB could increase fatty acid salt resistance without TolC, therefore, it was suggested that other genes were involved in the resistance regulated by EmrAB (Yoneda et al., 2022). We showed that the deletion of *emrAB* increased susceptibility to fatty acid salts, and the complementation of *emrAB* on the plasmid in the Δ*emrAB* strain reconstituted the resistance of the wild-type strain in *E. coli* (Table 3). However, the susceptibility of Δ*emrAB*Δ*tolC*, Δ*emrAB*Δ*tolC*/vector, and Δ*emrAB*Δ*tolC*/p*emrAB* strains did not change from that of Δ*tolC* strain, indicating that, unlike in *S. enterica*, EmrAB requires TolC for fatty acid salt efflux in *E. coli* (Table 3). EmrA and EmrB are 89.7 and 95.7% identical in their amino acid sequences between *E. coli* and *S. enterica* (Nishino et al., 2006). However, the difference in the structure of EmrAB between two species is unknown and it is difficult to predict the difference related to interaction with TolC from their amino acid sequences because not only changes in specific amino acids, but also the overall structure of EmrAB is involved in TolC interaction. On the other hand, it has been shown that the substrates for EmrAB are different between *E. coli* and *S. enterica* (Lomovskaya and Lewis, 1992; Nishino and Yamaguchi, 2001; Nishino et al., 2006), therefore, there may be difference in the structure of EmrAB between two species. It is also possible that there are other unknown outer membrane proteins in *S. enterica* that could be used by EmrAB.

Infecting bacteria into the host cells must be resistant to antimicrobial compounds. Fatty acid salts are host-derived antimicrobial molecules (Henderson et al., 2021). Therefore, each efflux pump has the function to efflux fatty acid salts to protect *E. coli* from accumulating these compounds.

In conclusion, we provide valuable insights on how multidrug efflux pumps confer fatty acid salt resistance in *E. coli*.

## Data availability statement

The original contributions presented in the study are included in the article/supplementary material, further inquiries can be directed to the corresponding author.

## Author contributions

TY, SY, and SI conceived the experiments. MH-N and KN designed the study. SY, SI, M-HN, and KN constructed strains. TY, SY, SI, M-HN, and KN analyzed data. TY and KN wrote the manuscript. KN supervised the project. All authors contributed to the article and approved the submitted version.

## Funding

This study was supported by a research grant from Kakenhi (18K19451, 21H03542, 21K16318, and 22K19831) from the Japan Society for the Promotion of Science, Core Research for Evolutional Science and Technology (JPMJCR20H9) from the Japan Science and Technology Agency, Japan Agency for Medical Research and Development, the Takeda Science Foundation, the Nippon Foundation-Osaka University Project for Infectious Disease Prevention, International Joint Research Promotion Program of Osaka University, and Research Program for CORE^2^-A lab, Network Joint Research Center for Materials and Devices and Dynamic Alliance for Open Innovation Bridging Human, Environment, and Materials from the Ministry of Education, Culture, Sports, Science and Technology of Japan.

## Conflict of interest

TY is employed by Novartis Pharmaceutical K.K which provided Ph.D. support to TY However, the company did not participate in study design, collection, analysis, and interpretation of data, and writing the manuscript. TY and SI were graduate students at the Graduate School of Pharmaceutical Sciences, Osaka University, and conducted research at SANKEN (The Institute of Scientific and Industrial Research), Osaka University. SY, MH-N, and KN were employed by SANKEN (The Institute of Scientific and Industrial Research), Osaka University, Osaka, Japan.

## Publisher’s note

All claims expressed in this article are solely those of the authors and do not necessarily represent those of their affiliated organizations, or those of the publisher, the editors and the reviewers. Any product that may be evaluated in this article, or claim that may be made by its manufacturer, is not guaranteed or endorsed by the publisher.
